# Bioactive Polymer Coatings Incorporating Curcumin and *Aloe vera* for Skincare Textiles

**DOI:** 10.3390/ma19214706

**Published:** 2026-11-05

**Authors:** Angela Danila, Ionela Cristina Voinea, Laura Chirilă, Miruna S. Stan, Rodica Roxana Constantinescu, Emil-Ioan Muresan

**Affiliations:** 1 Faculty of Industrial Design and Business Management, “Gheorghe Asachi” Technical University of Iasi, 29 Prof. Dr. Docent D. Mangeron Blvd, 700050 Iasi, Romania; angela.danila@academic.tuiasi.ro; 2 Centre for Research and Innovation in Textiles and Fashion Industry-SMART-Tex-IS, Corp TEX 4, 29 Prof. Dr. Docent D. Mangeron Blvd, 700050 Iasi, Romania; 3 Department of Biochemistry and Molecular Biology, Faculty of Biology, University of Bucharest, 91-95 Splaiul Independentei, 050095 Bucharest, Romania; miruna.stan@bio.unibuc.ro; 4 National Research and Development Institute for Textiles and Leather—INCDTP, 16 Lucrețiu Pătrășcanu, 030508 Bucharest, Romania; laura.chirila@incdtp.ro (L.C.); rodica.constantinescu@icpi.ro (R.R.C.); 5 “Cristofor Simionescu” Faculty of Chemical Engineering and Environmental Protection, “Gheorghe Asachi” Technical University of Iasi, 73 Prof. Dr. Docent D. Mangeron Blvd, 700050 Iasi, Romania; emil-ioan.muresan@academic.tuiasi.ro

**Keywords:** functional textiles, sodium alginate, curcumin, *Aloe vera*, curcumin release

## Abstract

Bioactive polymer coatings containing curcumin and *Aloe vera* were developed in a sodium alginate matrix and applied to woven cotton fabrics for skincare applications. Seven dispersions with different concentrations of curcumin, *Aloe vera*, and sodium alginate were applied by knife coating to cotton substrates pretreated with CaCl_2_ to promote in situ ionic crosslinking. The coated fabrics were evaluated for coating deposition, morphology, elemental composition, color, curcumin release, antibacterial activity, and in vitro cellular response. Coating add-on ranged from 9.44 to 17.73%, depending on the formulation composition, while SEM observations confirmed the presence of film-like polymer deposits on the fiber surfaces. EDX analysis detected elements associated with the alginate coating and CaCl_2_ pretreatment. Color intensity was strongly correlated with the yellow component b* (*r* = 0.976, *p* < 0.001), but was not significantly correlated with the add-on (*r* = 0.547, *p* = 0.204), which indicates that color intensity was not determined by the deposited mass. The strongest antibacterial activity was observed for the formulation containing 1.5% (*w*/*v*) sodium alginate, 0.5% (*v*/*v*) glycerol, 0.5% (*v*/*v*) Tween 20, 0.1% (*w*/*v*) curcumin and 1% (*v*/*v*) *Aloe vera*, with inhibition zones of 9.50 ± 0.87 mm against *E. coli* and 8.83 ± 0.58 mm against *S. aureus*. Although some samples produced no measurable inhibition zones, no bacterial growth was observed in the contact area beneath the specimens, indicating a satisfactory antibacterial effect according to SR EN ISO 20645:2004. The release of curcumin was initially faster and subsequently reached a plateau, and the kinetic parameters were consistent with a release controlled primarily by diffusion. Extracts from all coated fabrics were well tolerated by HaCaT keratinocytes, with no relevant effects on the investigated cellular parameters. These results support the potential of alginate coatings containing curcumin and *Aloe vera* for the development of bioactive cotton textiles intended for direct skin contact.

## 1. Introduction

Textile materials used in skincare are designed to come into direct contact with the skin and offer benefits that go beyond basic protection and comfort. Through suitable functionalization, textile substrates can serve as carriers for bioactive substances and provide moisturizing, antimicrobial, antioxidant, UV-protective, or controlled-release properties [1,2,3,4]. Current research increasingly focuses on incorporating plant-derived active compounds into biocompatible polymeric coatings, emulsions, or encapsulation systems to improve their stability and control their release at the skin–textile interface [1,2,3,4,5]. Cotton is particularly suitable for such applications because of its softness, breathability, absorbency, and compatibility with aqueous finishing processes [1,2,4,5].

The growing use of skincare textiles increasingly relies on plant-derived bioactive compounds that can provide complementary antimicrobial, antioxidant, anti-inflammatory, UV-protective, and skin-soothing effects [3,4,5,6,7]. The use of these compounds also supports the development of more sustainable textile finishing systems based on renewable active ingredients and biocompatible carrier materials [3,4,5,6,7]. Nevertheless, the direct application of many natural compounds may be limited by their low water solubility, sensitivity to processing and storage conditions, weak affinity for textile fibers, or uncontrolled release. Consequently, recent approaches have involved incorporating these compounds into polymer coatings, emulsions, microcapsules, or other delivery systems to enhance their stability, distribution, retention, and availability at the textile–skin interface [1,2,3,5,6,7].

Among the natural bioactive compounds investigated for textile functionalization, curcumin is particularly attractive because of its antimicrobial, antioxidant, anti-inflammatory, and UV-absorbing properties [3,8,9,10,11]. Curcumin and its derivatives have been incorporated into sol–gel coatings, polyurethane dispersions, and polymeric composites applied to cotton substrates, resulting in materials with antibacterial and other functional properties [8,9,10,11]. Nevertheless, its practical application is limited by its poor water solubility, susceptibility to light and oxidation, limited affinity for cellulose fibers, and potential loss during use. Incorporating curcumin into an appropriate polymeric matrix can improve its dispersion and retention on the textile surface while allowing more controlled release of the active compound [8,9,10,11].

*Aloe vera* is commonly used in skincare products because of its moisturizing, antimicrobial, antioxidant, anti-inflammatory, and skin-regenerating properties, which are associated with its polysaccharides, phenolic compounds, enzymes, and anthraquinone derivatives [2,12,13]. It has been incorporated into woven textiles, polymeric membranes, electrospun structures, and facial masks designed for skin-contact and cosmetic applications [2,12,13]. When used with polymeric carriers, *Aloe vera* can help retain moisture, support cellular compatibility, and provide local availability of bioactive compounds. Despite these advantages, its effectiveness is influenced by its concentration, processing conditions, and interactions with the carrier matrix and other active ingredients. Therefore, biological assessment of the final textile material is necessary [2,12,13].

Polymeric matrices are essential for incorporating and stabilizing natural bioactive compounds and enabling their controlled release from textile surfaces. Sodium alginate is particularly suitable for this purpose because of its biocompatibility, biodegradability, hydrophilic properties, and ability to form coatings and gels through ionic crosslinking with calcium ions [14,15,16]. Alginate-based systems have been used to incorporate both *Aloe vera* and curcumin, thereby helping to retain moisture and allowing the gradual release of active compounds [14,16,17]. However, their performance is influenced by polymer concentration, crosslinking conditions, coating homogeneity, and interactions among the incorporated components. Therefore, the entire formulation must be optimized and physicochemically characterized [14,15,16,17].

The number of studies combining curcumin and *Aloe vera* in the same bioactive material remains limited, and most have focused on wound-care applications. Previously investigated systems include coated bamboo-based dressings, PVA/*Aloe vera* hydrogel membranes, chitosan/*Aloe vera* films, and electrospun polymeric microfibers [18,19,20,21]. In the textile field, researchers have applied *Aloe vera*, chitosan, and curcumin to cotton via the exhaustion method, focusing on antimicrobial activity and wash durability [22]. In contrast, this study applies an active system, embedded in sodium alginate, to a textile substrate using a knife-coating technique. Furthermore, the concentrations of curcumin, *Aloe vera*, and sodium alginate were varied individually, starting from a reference formulation while keeping the other components constant, to assess the influence of the composition on the properties of the final material. Although these studies indicate that the two natural compounds are compatible and possess complementary biological properties, they mainly involve membranes, hydrogel dressings, or electrospun structures intended for use on damaged skin. In comparison, considerably less attention has been paid to incorporating both substances into an alginate coating applied directly to woven cotton for skincare applications and to comprehensively evaluating dispersion characteristics, coating deposition and morphology, curcumin release, antibacterial activity, and keratinocyte response.

For single-use textiles intended for short-term skin contact, the antimicrobial effect is not sufficient to determine their suitability for use. The assessment must also include the uniformity of the coating, the release of the active compound, and compatibility with skin cells [1,23,24]. Recent studies have highlighted the importance of combining traditional microbiological tests with assessments using keratinocytes or more sophisticated skin models to provide a better understanding of textile–skin interactions and detect possible cytotoxic or inflammatory effects [1,23,24,25]. This comprehensive approach is especially important for multicomponent natural formulations because their overall biological response cannot be predicted solely from the known properties of the individual ingredients.

Therefore, this study aimed to develop coatings based on sodium alginate, curcumin, and *Aloe vera*, and to apply the bioactive dispersion onto cotton fabrics intended for single-use, short-duration skin care products. Different compositions were investigated to determine the relationships among dispersion morphology, coating deposition and surface morphology, curcumin release, antibacterial activity against *E. coli* and *S. aureus*, and the response of human keratinocytes. This approach provides a basis for selecting multifunctional textile substrates that are well-tolerated in vitro and intended for direct contact with the skin.

## 2. Materials and Methods

### 2.1. Materials

Curcumin powder (for synthesis), polysorbate 20 (Tween 20), and sodium alginate were purchased from Merck KGaA (Darmstadt, Germany). Tween 20 was used as a nonionic surfactant, while sodium alginate served as the biopolymer matrix. Vegetable glycerin (99.5% purity, Herbavit, Oradea, Romania) was used as a humectant and plasticizing agent, while organic *Aloe vera* powder (Mayam, Oradea, Romania) was used as a skincare active ingredient. The textile substrate was a plain-weave 100% cotton fabric with a mass per unit area of 130 g/m^2^, provided by a local textile finishing company.

### 2.2. Preparation of Bioactive Alginate-Based Dispersions

Seven bioactive alginate-based dispersions containing curcumin and *Aloe vera* were prepared using the formulations presented in Table 1. To improve its dispersion, curcumin was first dissolved in absolute ethanol to prepare a stock solution with a concentration of 50 mg/mL. The sodium alginate stock solution (4% *w*/*v*) was prepared by dissolving sodium alginate in distilled water under continuous stirring for 2 h at room temperature. The other components were added sequentially in the following order: glycerin, Tween 20, ethanolic curcumin solution, and *Aloe vera* powder. The mixture was mechanically stirred for 5 min after each addition to ensure complete homogenization and for 10 min after the final addition. The use of Tween 20 limited the precipitation and agglomeration of curcumin upon the addition of the ethanolic solution to the aqueous phase by solubilizing it within the hydrophobic core of the surfactant micelles [26,27,28]. Furthermore, the surfactant Tween 20 facilitated the wetting and dispersion of the *Aloe vera* powder and reduced the surface tension of the dispersion, thereby enabling its uniform application onto the surface of the textile substrate [29].

Sample 2 was considered the reference formulation. The concentration of curcumin was varied in samples 2, 3, and 4; the concentration of *Aloe vera* in samples 1, 2, and 5; and the concentration of sodium alginate in samples 2, 6, and 7. The concentrations of glycerin and Tween 20 were the same in all formulations (0.5% *v*/*v*).

### 2.3. Application of Dispersions to the Textile Substrate

Prior to functionalization, the cotton fabric was preliminarily prepared by two successive processes consisting of alkaline scouring and bleaching. The specimens were then rinsed repeatedly with hot and cold water for 10 min and dried at room temperature. The cotton specimens were subsequently immersed in a 2% (*w*/*v*) CaCl_2_ aqueous solution for 30 min to incorporate Ca^2+^ ions into the fibrous structure and enable the subsequent in situ ionic crosslinking of sodium alginate [30]. The bioactive alginate-based dispersions were applied to the CaCl_2_-treated cotton specimens by knife coating. The coated specimens were placed in open Petri dishes and allowed to dry for 24 h under controlled atmospheric conditions of 20 ± 2 °C and 65 ± 4% relative humidity. During drying, the specimens were protected from light to minimize curcumin photodegradation.

### 2.4. Characterization of the Bioactive Textiles

#### 2.4.1. Optical Microscopy

The seven dispersions were analyzed using an optical microscope (A.KRÜSS Optronic GmbH, Hamburg, Germany) equipped with a COOLPIX P5100 digital camera (Nikon Corporation, Tokyo, Japan) at 40× magnification.

#### 2.4.2. Add-On

The add-on was calculated according to Equation (1):

(1) $$Add-on\;(\%)\;=\frac{w_{f}-w_{i}}{w_{i}}\times 100$$

where $w_{i}$ is the mass of the textile specimen determined before pre-treatment with CaCl_2_, and $w_{f}$ is the mass of the dried specimen after the application of the dispersion.

A correlation was established among the add-on (%), the applied (wet) dispersion weight, and the dry dispersion weight to evaluate how the amount of dispersion applied to the fabric relates to the amount effectively retained on the substrate after drying.

The dispersion weight (g) was determined by weighing the amount of dispersion applied to the fabric. Any dispersion remaining on the doctor blade after coating was collected and redeposited onto the textile support to minimize handling losses and ensure that the reported value closely reflected the total amount transferred to the fabric.

The mass of the dry dispersion retained on the material was calculated as the difference w_f_ − w_i_. The solids content (SC) was determined gravimetrically by drying a known quantity of the dispersion in a beaker, and was calculated using Equation (2):

(2) $$SC\;(\%)=\frac{\left(m_{2}-m_{b}\right)}{\left(m_{1}-m_{b}\right)}\;\times 100$$

where $m_{1}$ is the mass of the container with the wet dispersion, $m_{2}$ is the mass of the container with the oven-dried dispersion, and $m_{b}$ is the mass of the empty container.

The add-on values were determined once for each formulation (*n* = 1).

#### 2.4.3. Scanning Electron Microscopy and Energy-Dispersive X-Ray Spectroscopy (SEM-EDX)

Morphological and elemental analyses were performed using an FEI Quanta 200 scanning electron microscope (FEI Company, Hillsboro, OR, USA) equipped with an EDAX Genesis EDS detector (AMETEK Inc., Mahwah, NJ, USA). EDX data were acquired and processed using EDAX Genesis software, 5.2 version.

#### 2.4.4. Biocompatibility Assessment

Human keratinocytes (HaCaT cell line) were cultured in Dulbecco’s Modified Eagle Medium (DMEM; Sigma-Aldrich, St. Louis, MO, USA) supplemented with 10% fetal bovine serum (FBS; Gibco, Grand Island, NY, USA) at 37 °C in a humidified atmosphere containing 5% CO_2_. HaCaT cells were exposed to conditioned media obtained from the different tested fabrics to assess their biocompatibility. Small fabric pieces (0.3 cm × 0.3 cm) were first sterilized by exposure to UV light for 2 h, immersed in 1 mL of complete DMEM, and incubated under constant shaking at 240 rpm for 72 h.

Cell viability was determined using the MTT assay [3-(4,5-dimethylthiazol-2-yl)-2,5-diphenyltetrazolium bromide], based on the ability of cellular enzymes to reduce MTT to a purple formazan product. Nitric oxide (NO) production was measured using the Griess colorimetric assay by recording the absorbance at 550 nm after mixing the culture medium and reagent in equal volumes. To evaluate membrane damage following exposure to the fabric extracts, lactate dehydrogenase (LDH) release was quantified. In this assay, 50 µL of cell culture supernatant was mixed with an equal volume of dye and catalyst solution from the Cytotoxicity Detection Kit (Roche, Mannheim, Germany) and incubated for 30 min at room temperature. The absorbance was then measured at 490 nm using a FlexStation 3 microplate reader (Molecular Devices, San Jose, CA, USA).

All data were expressed as mean value ± standard deviation (SD) of three different experiments. The results were expressed relative to the untreated cotton fabric used as a control. Statistical analysis was performed using one-way ANOVA followed by Tukey’s post hoc test using GraphPad Prism 11 (GraphPad software Inc., La Jolla, CA, USA), and results with *p*-values below 0.05 were considered statistically significant.

Morphological changes in the cytoskeleton following exposure to the fabric extracts were assessed using phalloidin conjugated with FITC (fluorescein isothiocyanate). For this purpose, the cell culture medium was aspirated, and the cells were fixed in the wells with 100 µL/well of 4% paraformaldehyde solution in PBS for 20 min at room temperature. The membranes of the fixed cells were permeabilized with 100 µL/well of 0.1% Triton X-100 and 1.2% BSA in PBS for 1 h. Traces of detergent were then removed by three washes with PBS, and the cells were incubated for 1 h with 130 µL of phalloidin-FITC solution (stock concentration: 20 µg/mL) to stain the actin filaments. Subsequently, the cells were washed three times with PBS and visualized using an Olympus IX71 inverted fluorescence microscope (Olympus, Tokyo, Japan).

#### 2.4.5. Antibacterial Activity

The antibacterial activity of the functionalized textile samples was evaluated against *S. aureus* ATCC 6538 (Gram-positive) and *E. coli* ATCC 11229 (Gram-negative) using the agar diffusion method in accordance with SR EN ISO 20645:2004. Fresh pure cultures of both bacterial strains were used, and nutrient agar served as the culture medium. The textile specimens were placed on two-layer agar plates. The lower layer consisted of 10 ± 0.1 mL of sterile, bacteria-free culture medium. To prepare the upper layer, 150 mL of culture medium, cooled to approximately 45 °C, was inoculated with 1 mL of bacterial suspension at a concentration of 1–5 × 10^8^ CFU/mL. After thorough mixing, 5 ± 0.1 mL of the inoculated medium was added to each Petri dish and allowed to solidify.

Circular textile specimens with a diameter of 20 ± 5 mm were placed on the surface of the inoculated medium and incubated at 37 °C for 24 h. Antibacterial activity was evaluated by examining bacterial growth in the contact area beneath the textile specimens and measuring the width of any inhibition halo extending beyond their edges. The inhibition-zone width (H) was calculated using the following equation:

(3) H=(D−d)/2

where H is the width of the inhibition halo outside the textile specimen (mm), D is the total diameter of the textile specimen and the surrounding inhibition zone (mm), and d is the diameter of the textile specimen (mm). Thus, the reported H values exclude the specimen diameter. When no external inhibition halo was observed, H was recorded as 0 mm, and bacterial growth beneath the specimen was assessed separately.

All measurements were performed in triplicate, and the results were expressed as the mean ± standard error of the mean.

#### 2.4.6. Color Analysis

The application of dispersions containing different amounts of curcumin resulted in colored textile substrates. The color characteristics of the functionalized fabrics were evaluated using the CIELAB parameters L*, a*, and b*. Color strength (K/S) was calculated from the reflectance measured at 430 nm, corresponding to the characteristic absorption maximum of curcumin, using the Kubelka–Munk equation [30]:

(4) $$\frac{K}{S}=\frac{{\left(1-R\right)}^{2}}{2R}$$

where R  is the reflectance, K  is the absorption coefficient, and S  is the scattering coefficient. The L*, a*, b*, and K/S values represent the average of five readings for each sample.

Pearson correlation coefficients were calculated to examine the relationships among the CIELAB parameters, color strength, and add-on, and the resulting correlation matrixwas presented in Table 2. The statistical significance of the correlation coefficients was evaluated using the *t*-test for the Pearson correlation coefficient (*n* = 7), at a threshold of *p* < 0.05.

#### 2.4.7. Kinetics of Curcumin Release from the Textile Substrate

The textile specimens treated with the seven dispersions were immersed in 50 mL of physiological saline and maintained at 32 °C for 20 h. At predetermined time intervals, 5 mL aliquots were withdrawn and analyzed spectrophotometrically using a Camspec M501 UV-VIS spectrophotometer. After each withdrawal, the sampled volume was replaced with 5 mL of physiological saline. Release profiles were determined once for each formulation (*n* = 1). The curcumin concentration was determined based on a standard curve obtained using curcumin solutions of 1, 4, and 6 µg/mL, prepared by diluting a 1 mg/mL ethanolic stock solution in physiological saline (y = 0.0884x, R^2^ = 0.922, where y is the absorbance and x is the curcumin concentration (µg/mL)). The cumulative amount of released curcumin was corrected for the curcumin removed during previous sampling, using Equation (5).

(5) $$M_{t\left(n\right)}=V_{0}\times C_{n}+V_{s}\times (C_{1}+C_{2}+\cdots +C_{n-1})$$

where $V_{0\;}$ (50 mL) is the volume of the release medium, $V_{s}$ (5 mL) is the volume sampled, and $C_{n}$ (µg/mL) is the concentration of curcumin at time t_n_.

To investigate the mechanism of curcumin release from the polymer coating, the experimental data were fitted to the Higuchi and Korsmeyer–Peppas models [31,32].

Higuchi model

(6) $$Q_{t}=k_{H}\cdot t^{\frac{1}{2}}+b$$

where $k_{H}$ is the Higuchi constant, t is time (h), and b is the constant term.

Korsmeyer–Peppas model

*M_t_*/*M*_∞_ = *k* · *t_n_* (7)

where *k* is the Peppas release rate constant, *t* is time (h), *M_t_* is the amount of biologically active compound released at time t (µg), *M_∞_* is the initial amount of curcumin in the sample (µg), calculated as the product of the mass of the dry dispersion deposited on the sample and the fraction of curcumin within the non-volatile components of the dispersion, and *n* is the exponent characterizing the release mechanism of the biologically active compound.

The optimal model was selected according to the Akaike criterion (AIC) [33]:

(8) $$AIC=n\times ln(\frac{RSS}{n})+2p$$

where n is the number of experimental points, p is the number of fitted model parameters, RSS is the residual sum of squares between the experimental and model-predicted values.

Due to the limited number of experimental data points, the corrected Akaike criterion (*AICc*) was also calculated [34]:

(9) $${AIC}_{C}=AIC+\frac{2p(p+1)}{n-p-1}$$

Modeling was performed using 4 experimental data points (1–2.5 h), with two parameters adjusted for each model (*p* = 2). For both models, the *RSS* was calculated based on the experimental release values (%). The model with the lowest *AICc* value was considered optimal, while a difference of *ΔAICc* < 2 was deemed insufficient to distinguish between the models [34].

## 3. Results

### 3.1. Visual Appearance of the Treated Textile Samples

**Figure 1:**
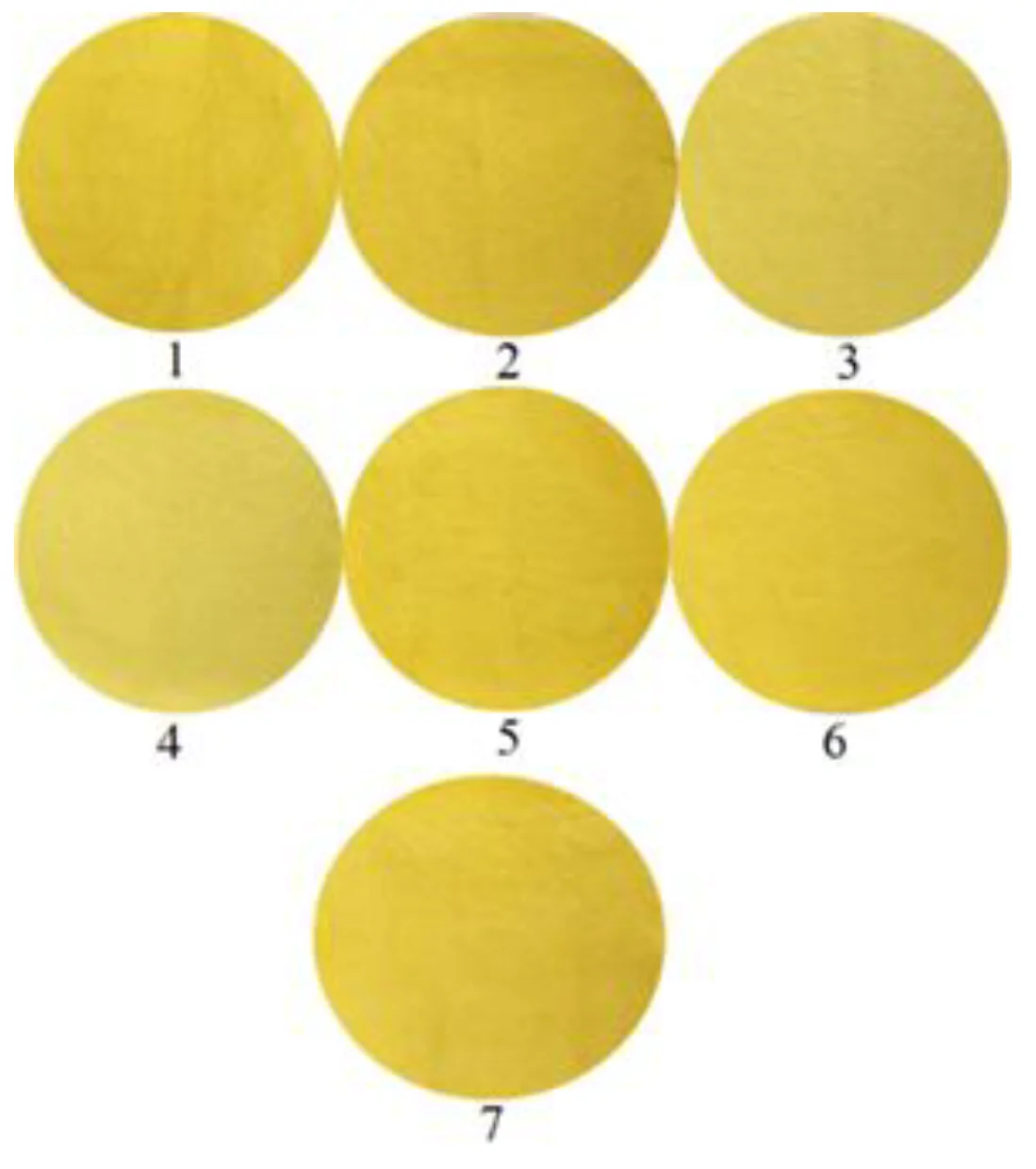
Images of treated textile materials.

Figure 1 shows the cotton textile samples coated with alginate-based dispersions containing curcumin and *Aloe vera*.

All treated samples exhibited the characteristic yellow coloration of curcumin, confirming the deposition of the bioactive alginate-based dispersions on the cotton substrate. Differences in color intensity were visible among the samples. Samples 3 and 4 showed a lighter yellow shade, consistent with their lower curcumin concentrations of 0.05% and 0.02%, respectively, compared with 0.1% in the other formulations. The differences observed among samples containing the same curcumin concentration may be related to variations in the amount and distribution of the coating retained on the textile surface. These aspects are examined further through add-on measurements and color analysis.

### 3.2. Optical Microscopy

**Figure 2:**
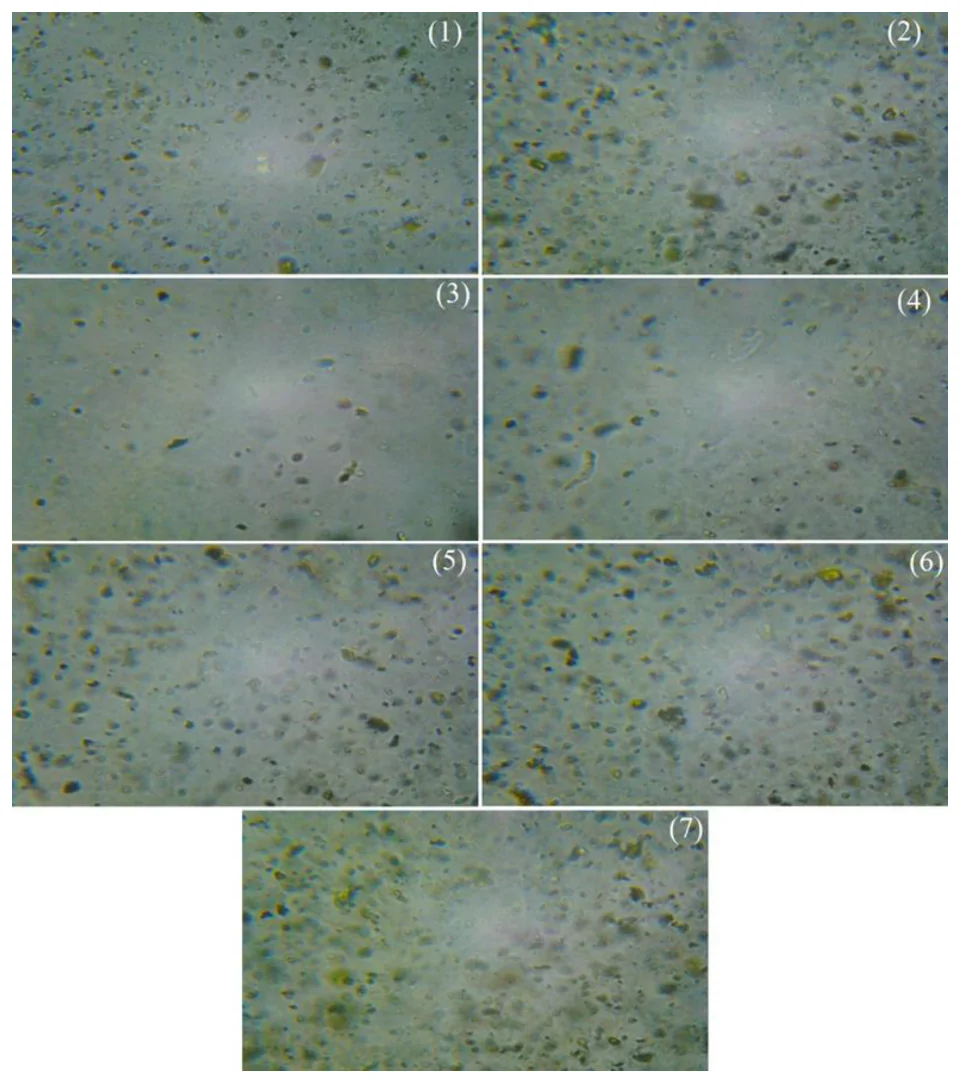
Optical micrographs of the bioactive alginate-based dispersions at the magnification of 40×.

The optical micrographs of the seven bioactive alginate-based dispersions are presented in Figure 2.

The micrographs show yellow regions attributed to curcumin that appear to be distributed throughout the alginate matrix. Overall, curcumin appears to be relatively uniformly dispersed, although some differences in the distribution of the visible regions can be observed among the formulations. No extensive agglomeration or clear phase separation was observed at the investigated magnification, indicating satisfactory incorporation of curcumin into the prepared dispersions. The satisfactory incorporation of curcumin into the prepared dispersions can be attributed to the stabilizing effect of the surfactant, Tween 20 [26,28].

### 3.3. Add-On

**Figure 3:**
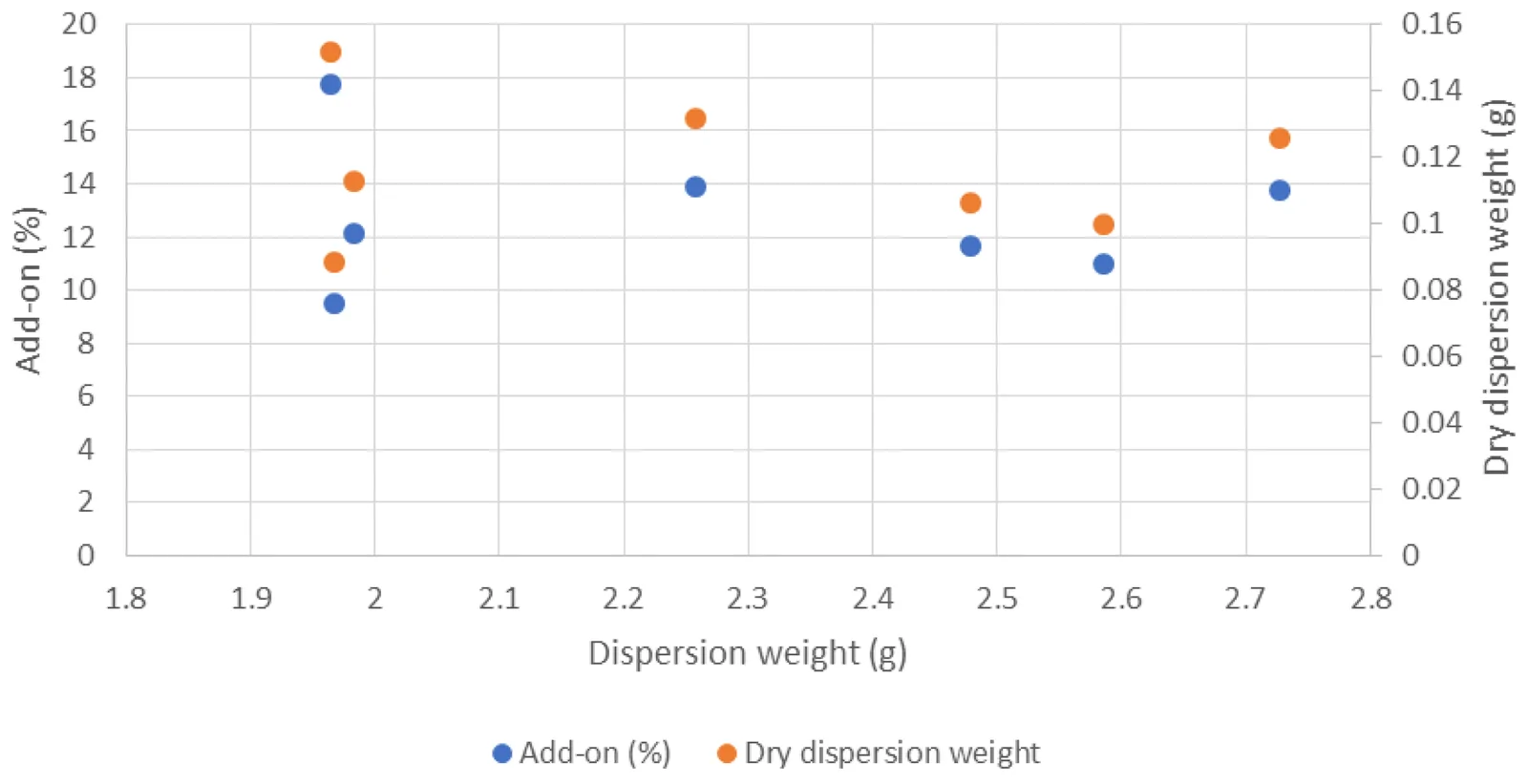
Relationship between dispersion weight, dry dispersion weight and add-on.

The percentage increase in the mass of the material after treatment was calculated according to Equation (1). The relationships among add-on (%), applied (wet) dispersion weight (g), and dry dispersion weight (g) are presented in Figure 3.

Based on the data presented in Figure 3, no clear correlation was observed between the amount of dispersion applied to the material and the add-on value: the add-on values varied independently of the increase in the weight of the applied dispersion, without a consistent trend over the studied range. In contrast, the dry dispersion weight was significantly lower than the initially applied wet dispersion weight, confirming the high water content of the dispersion. The solids content was approximately 3.5–4.8%, indicating that, after drying, only a small fraction of nonvolatile compounds remained on the textile material. Based on the results obtained, the add-on is not directly proportional to the mass of the applied wet dispersion, as it also depends on the solids fraction of each formulation and the initial mass of the textile substrate. In the series involving sodium alginate (samples 2, 6, and 7), the add-on increased with the sodium alginate concentration (9.44%, 12.13%, and 13.85%). The add-on values also include the contribution of the calcium chloride remaining in the material after pre-treatment, since the initial mass (w_i_) was determined before the CaCl_2_ pre-treatment, and the samples were not rinsed.

### 3.4. SEM and EDX Analysis

**Figure 4:**
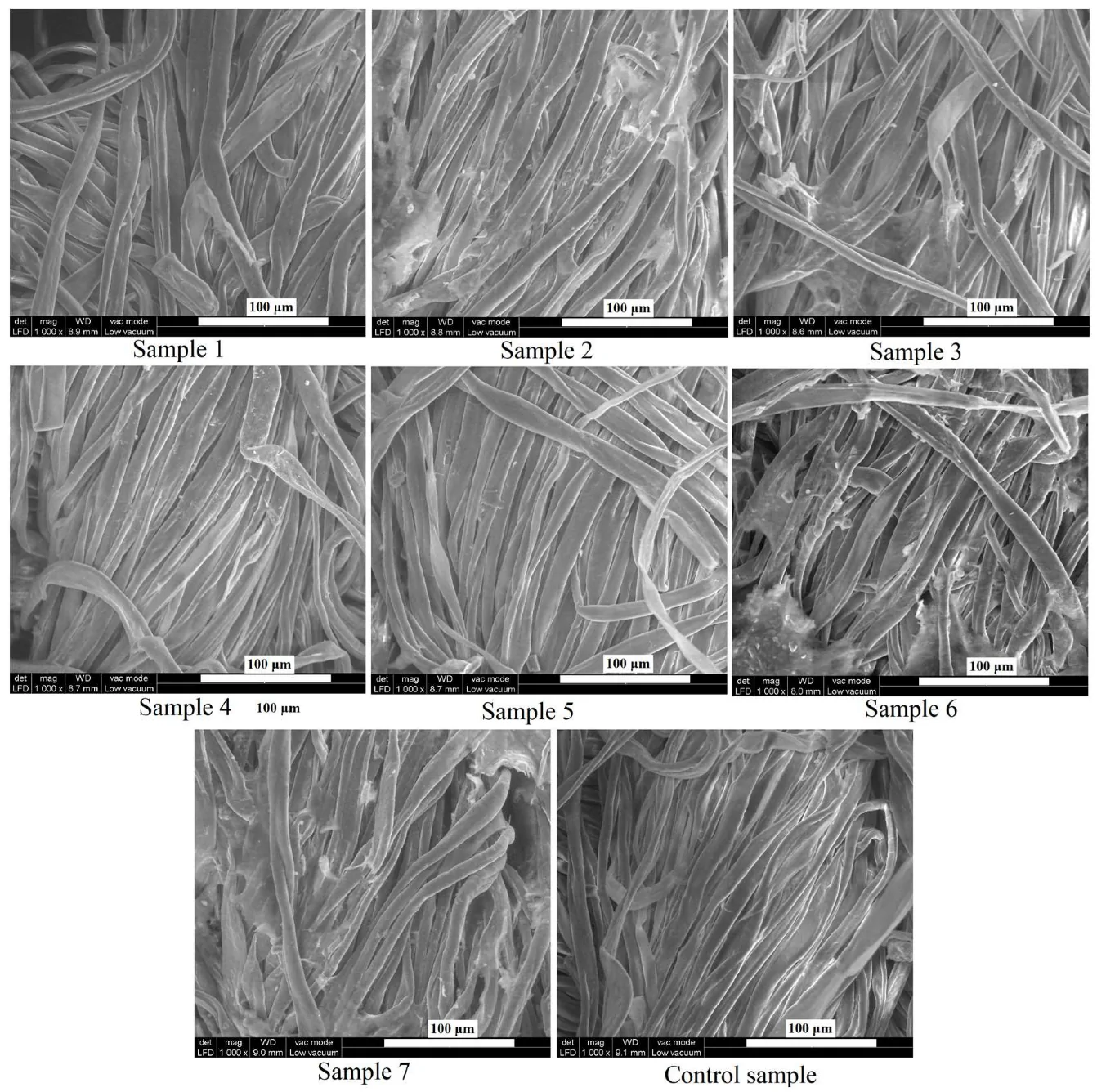
SEM images of the untreated cotton reference (M) and cotton fabrics functionalized with the bioactive polymer formulations (samples 1–7), recorded at 1000× magnification.

**Figure 5:**
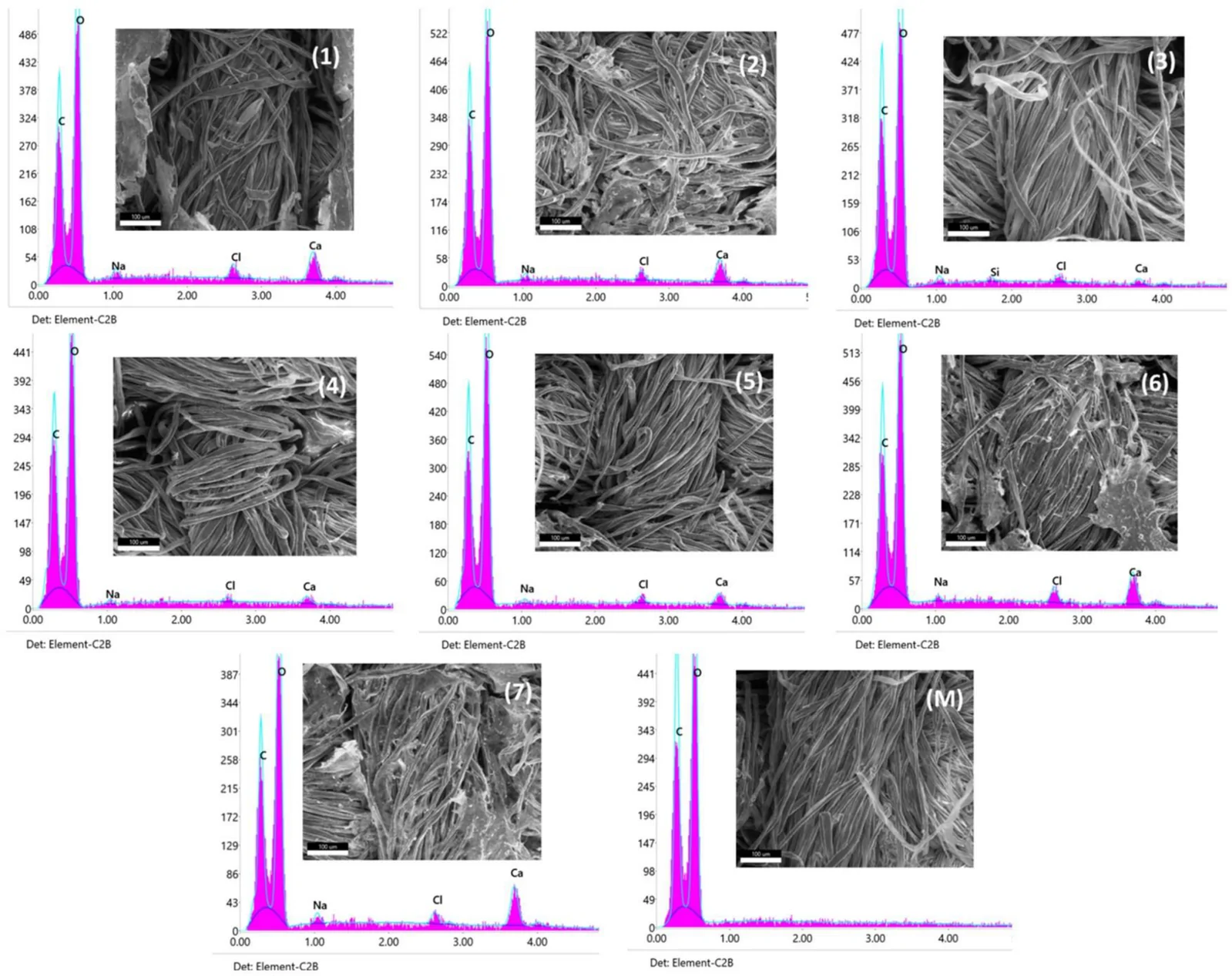
EDX spectra of untreated cotton (M) and functionalized textile samples (1–7), recorded at 500× magnification.

The SEM images of the untreated cotton reference and the coated textile samples, recorded at 1000× magnification, are presented in Figure 4. The EDX spectra of these samples, acquired from larger areas at 500× magnification, are shown in Figure 5.

The untreated cotton fabric exhibited its characteristic fibrous morphology, with clearly distinguishable individual fibers, longitudinal surface features, and relatively open interfiber spaces. After functionalization, deposits and film-like regions attributable to the polymer coatings were observed on the fiber surfaces. In some areas, the deposited material partially covered the fibers and formed bridges between adjacent fibers, indicating retention of the coating within the textile structure. Differences in the amount and distribution of the deposited material were observed among the formulations. Similar changes in surface morphology, including the formation of polymer layers on treated cotton fibers, have been reported for cotton substrates coated with curcumin-containing polymer formulations [35].

The untreated cotton fabric showed predominantly carbon and oxygen, as expected for a cellulosic substrate. In contrast, Na, Ca, and Cl were detected in all coated samples. The presence of Na was attributed to sodium alginate, whereas Ca and Cl were attributed to the CaCl_2_ pretreatment. Ca^2+^ ions may also participate in the ionic crosslinking of the deposited alginate matrix. The use of CaCl_2_-pretreated textile substrates to promote the in situ crosslinking of subsequently deposited sodium alginate has also been reported [36]. Because the specimens were not rinsed after CaCl_2_ treatment, residual calcium chloride may have remained within the textile structure. Thus, the signals indicating the presence of Ca and Cl may, in part, reflect residual CaCl_2_, and the EDX results point to the presence of these elements at the surface, rather than providing direct evidence of alginate cross-linking.

### 3.5. In Vitro Cellular Response to Textile Extracts

Before examining the antimicrobial performance of the coated textiles, we assessed the response of keratinocytes to extracts obtained from cotton fabrics coated with bioactive alginate-based dispersions containing curcumin and *Aloe vera*. Because keratinocytes are the predominant cell type in the epidermis, their response provides a relevant indication of the biocompatibility of materials intended for skincare applications. For this purpose, several complementary markers were analyzed, including metabolic activity, membrane integrity, and nitric oxide release, allowing the evaluation of general cell health and early signs of cytotoxicity after 24 h of exposure to the fabric extracts.

**Figure 6:**
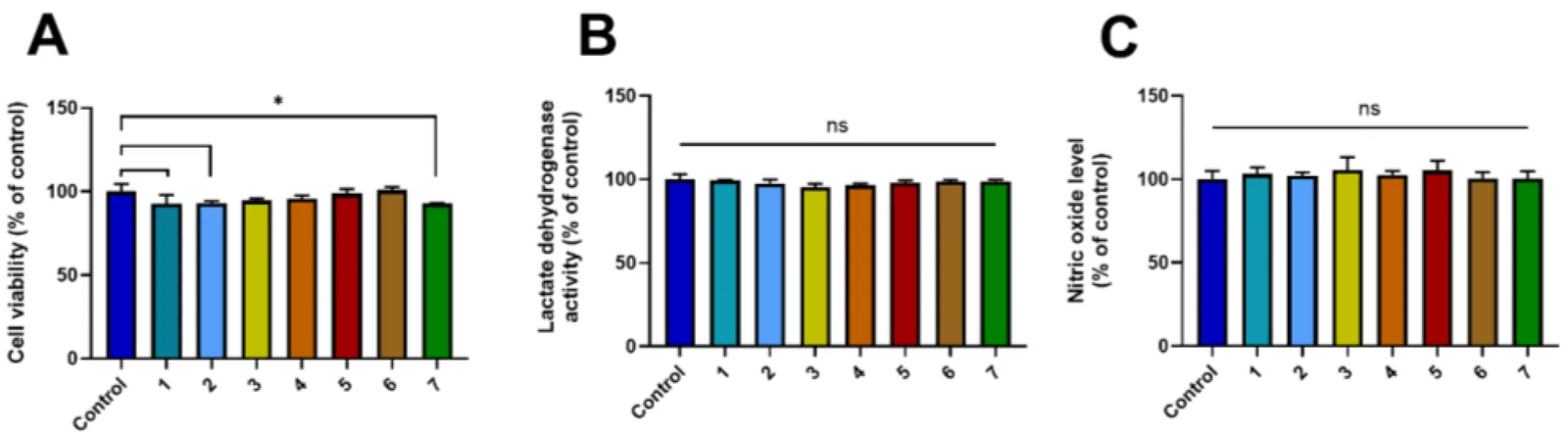
Biocompatibility profile of cotton fabrics following 24 h incubation with human keratinocytes (HaCaT cells) in terms of cell viability (**A**), lactate dehydrogenase (**B**) and nitric oxide (**C**) release. Results are calculated and represented as mean ± standard deviation (n = 3), normalized to cells treated with extracts from untreated fabric (Control). * *p* < 0.05, ns—no statistically significant differences were observed between treated samples and control.

Exposure of HaCaT keratinocytes to extracts obtained from the coated cotton fabrics for 24 h did not indicate any harmful cellular effects (Figure 6). The cells maintained high viability across all tested extracts, with values closely matching those of the untreated fabric control, suggesting that the materials were well tolerated. LDH release remained at baseline levels, indicating that cell membrane integrity was preserved. Consistent with these observations, nitric oxide release was also comparable to that of the control, indicating no detectable increase in the investigated inflammatory marker. Taken together, these results indicate that the treated fabrics did not induce cytotoxic effects under the investigated experimental conditions.

**Figure 7:**
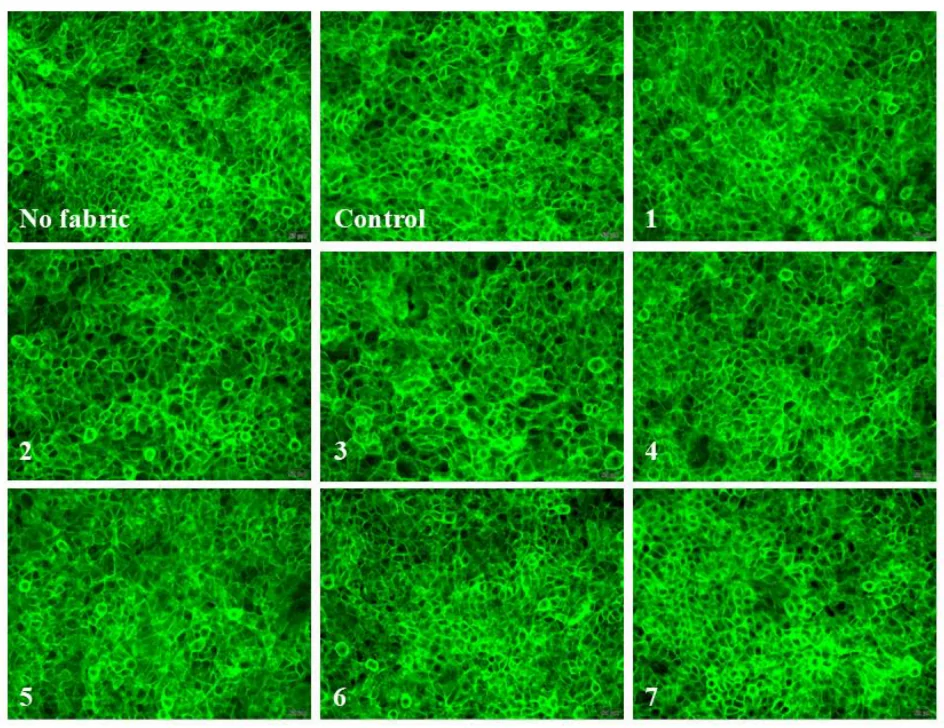
Representative fluorescence microscopy images showing the actin cytoskeleton (labeled with phalloidin-FITC in green) after 24 h of incubation of human keratinocytes (HaCaT cells) with different cotton fabrics. All fluorescence images were captured using a 20× magnification.

Fluorescence imaging of the stained actin cytoskeleton (Figure 7) provided further evidence that the fabric extracts did not induce cellular damage after 24 h of exposure. Keratinocytes maintained a well-defined actin network, with filament organization comparable to that observed in the control wells. Cells exposed to the extract obtained from the untreated cotton fabric (control) showed no alterations in cytoskeletal structure. Across all tested samples, the cells preserved their typical morphology, and their behavior was not affected by exposure to extracts obtained from cotton fabrics coated with alginate dispersions containing curcumin and *Aloe vera*.

The biocompatibility results obtained in this study are consistent with previous findings for cotton fabrics treated with *Aloe vera* or curcumin, which were reported to be well tolerated in skin-related applications. Most existing studies focus on antimicrobial or functional performance and provide only limited cytotoxicity data; nevertheless, they indicate that these natural bioactive compounds do not induce harmful cellular effects [32,37,38]. In the present study, the evaluation provided a more detailed view of the keratinocyte response to the coated textiles, showing preserved cell viability, stable LDH and nitric oxide release levels, and unchanged actin cytoskeleton organization after exposure to the fabric extracts. These findings are consistent with the reported biocompatibility of *Aloe vera* and curcumin and indicate that the alginate-based coatings containing both bioactive compounds were well tolerated by epidermal cells.

The good biocompatibility observed in the cotton samples may be related to the combined effects of the different components in the coating. Although Tween 20 has been reported to cause cytotoxic effects at certain concentrations [39], its presence in the formulation did not appear to negatively affect cell compatibility under the conditions tested. One possible explanation is that the alginate-based coating influences the release of Tween 20, limiting the amount available to the cells. In addition, curcumin and *Aloe vera* may contribute to the overall biological response due to their reported antioxidant and bioactive properties.

Curcumin may offer additional benefits due to its antioxidant and anti-inflammatory properties. However, some cases of allergic contact dermatitis following topical exposure to curcumin have been reported [40,41], suggesting that individual skin sensitivity should also be taken into account. It is important to note that the absence of cytotoxic effects does not necessarily exclude the possibility of allergic reactions, as these involve different biological mechanisms. For this reason, nitric oxide release was evaluated as an indicator of the inflammatory response to the developed cotton materials. All these findings provide useful preliminary evidence regarding the biological response to the materials and support further investigation of their potential use in skincare cosmetic applications.

### 3.6. Antibacterial Activity

**Figure 8:**
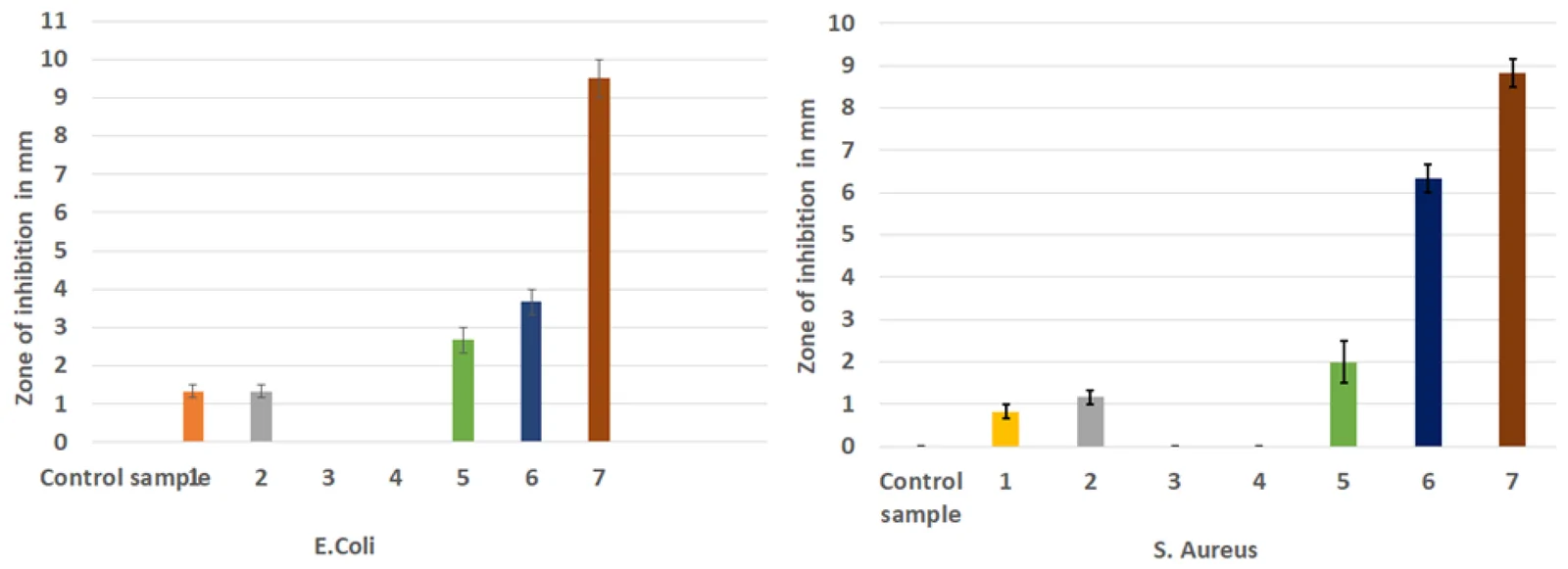
Inhibition zones of the untreated cotton control and cotton fabrics functionalized with bioactive alginate-based dispersions (samples 1–7) against *E. coli* and *S. aureus*. Values are expressed as the mean ± standard error of the mean (*n* = 3).

**Figure 9:**
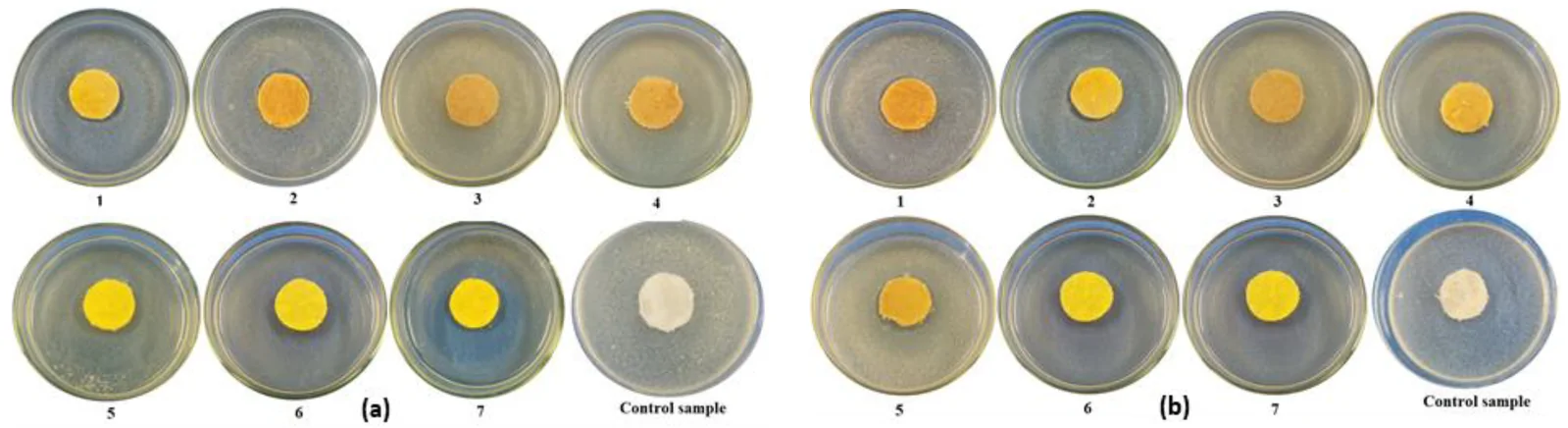
Representative agar diffusion plates showing the antibacterial activity of the untreated cotton control and functionalized cotton fabrics (samples 1–7) against *E. coli* (**a**) and *S. aureus* (**b**).

The antibacterial activity of the functionalized cotton fabrics against *E. coli* ATCC 11229 and *S. aureus* ATCC 6538 is presented in Figure 8, while representative images of the Petri plates are shown in Figure 9. Overall, sample 7 exhibited the strongest activity against both bacterial strains, with inhibition zones of 9.50 ± 0.87 mm against *E. coli* and 8.83 ± 0.58 mm against *S. aureus*. This was followed by sample 6, which was particularly effective against *S. aureus*, with an inhibition zone of 6.33 ± 0.58 mm. Because samples 2, 6, and 7 contained the same concentrations of curcumin and *Aloe vera* but increasing sodium alginate concentrations of 1%, 1.25%, and 1.5%, respectively, the stronger activity of samples 6 and 7 may be associated with improved deposition and retention of the bioactive compounds within the coating and their subsequent availability at the textile–agar interface.

Samples 1 and 2, containing 0.1% curcumin and 0.5% or 1% *Aloe vera*, respectively, produced similar inhibition zones, indicating that this increase in *Aloe vera* concentration had only a limited effect. By contrast, sample 5, containing 1.5% *Aloe vera*, showed larger inhibition zones against both strains. The antibacterial activity of *Aloe vera*-containing coatings on cotton fabrics has also been reported previously [42,43]. A comparison of samples 2, 3, and 4 highlighted the influence of curcumin concentration on antibacterial activity. These samples contained the same concentrations of *Aloe vera* and sodium alginate, whereas the curcumin concentration decreased from 0.1% in sample 2 to 0.05% and 0.02% in samples 3 and 4, respectively.

While sample 2 produced measurable inhibition zones against both bacterial strains, no external inhibition halos were observed for samples 3 and 4. This may be associated with their lower curcumin concentrations and limited diffusion of the bioactive compounds into the surrounding agar [44]. Nevertheless, no bacterial growth was observed beneath these specimens, indicating a satisfactory antibacterial effect according to SR EN ISO 20645:2004 [45]. The observed inhibition was localized to the textile–agar contact area. However, this finding alone does not establish the underlying mechanism.

The possible effects of alginate concentration on coating deposition, bioactive-compound retention, and availability at the textile–agar interface remain tentative explanations, as their individual contributions to antibacterial performance were not directly established experimentally. Overall, no consistent difference in antibacterial activity was observed between the two bacterial strains.

### 3.7. Color Analysis

The CIELAB color parameters (L*, a*, and b*) and color strength (*K/S*) were determined for the textile substrates treated with the seven dispersions. To examine the relationships among the CIELAB parameters, *K/S*, and add-on, the results were analyzed using a Pearson correlation matrix. The correlation between color strength and add-on (Table 2) was not statistically significant (*r* = 0.547, *p* = 0.204), which indicates that color strength was not determined by the total mass retained on the textile substrate after drying. This result may be explained by the incorporation of curcumin within the dispersed system, which may limit its availability at the surface of the textile substrate. The strongest positive, statistically significant correlation was observed between color strength and b* (*r* = 0.976, *p* < 0.001), indicating that the yellow component became more pronounced as color strength increased.

This trend was particularly evident for samples 2–4, in which higher curcumin concentrations resulted in higher *K/S* and b* values. Similar color parameters for cotton fabrics functionalized with curcumin have also been reported in the literature [8,46].

A significant negative correlation was observed between color intensity and L* (*r* = −0.766, *p* = 0.045), indicating that samples with higher color intensity were also darker. The strong negative correlation between L* and a* (*r* = −0.976, *p* < 0.001) reflects the fact that the samples with the lowest curcumin concentrations (samples 3 and 4) were the lightest and exhibited negative a* values. The correlations between the add-on and the L*, a*, and b* parameters were not statistically significant (*p* > 0.05). Given the small number of formulations (*n* = 7), these correlations should be interpreted as trends for the systems investigated.

### 3.8. Curcumin Release Kinetics from Coated Textile Materials

The time-dependent release of curcumin from the textile substrate was determined at predetermined intervals of 1 h, 1.5 h, 2 h, 2.5 h, 3 h, 3.5 h, and 20 h.

**Figure 10:**
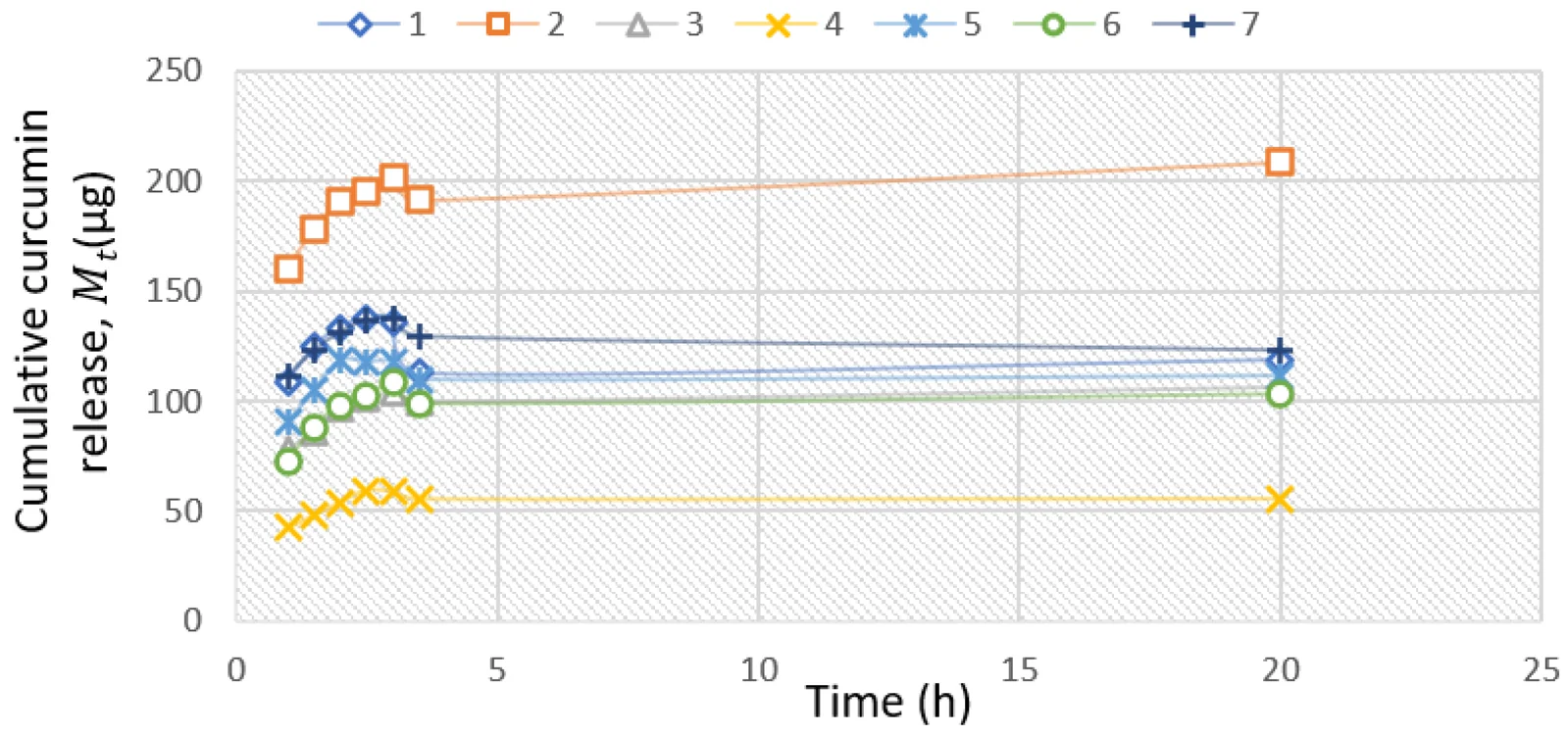
Curcumin release profile.

The amount of curcumin released over time was determined based on the calibration equation obtained from the standard curve described in Section 2.4.7. Since the calibration curve passes through the origin, any error in the slope would alter all concentrations by the same factor. Consequently, the exponent n, the R^2^ coefficients, and the ΔAICc values used for model selection are not influenced by the slope of the calibration curve. After approximately 3 h, the curcumin remaining in the material appeared to be more strongly retained within the polymer matrix, resulting in a lower release rate. This behavior suggests that a release plateau was approached, but does not necessarily indicate the absence of curcumin in the material. The curcumin release profile is presented in Figure 10. Although the release profile was monitored for up to 20 h, kinetic modeling was performed only for the initial 2.5 h period, during which a clear increase in the amount of curcumin released was observed. After 3 h, the corrected cumulative amount decreased for all samples (at 3.5 h), possibly due to the chemical instability of curcumin in an aqueous environment [47]. All release values within the modeled interval were below 60%, thus falling within the applicability range of the Korsmeyer–Peppas model.

**Figure 11:**
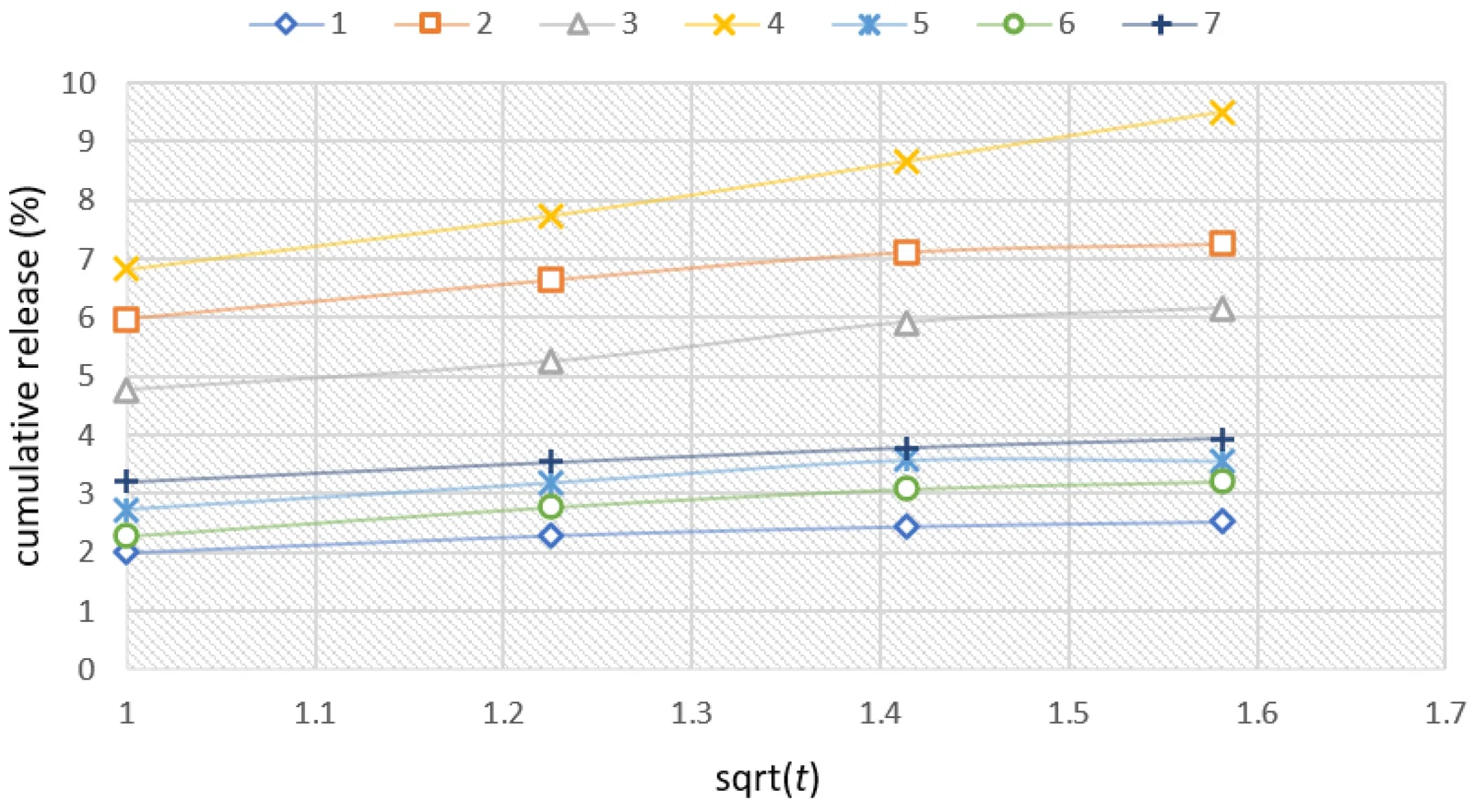
Release kinetic profile according to the Higuchi model.

**Figure 12:**
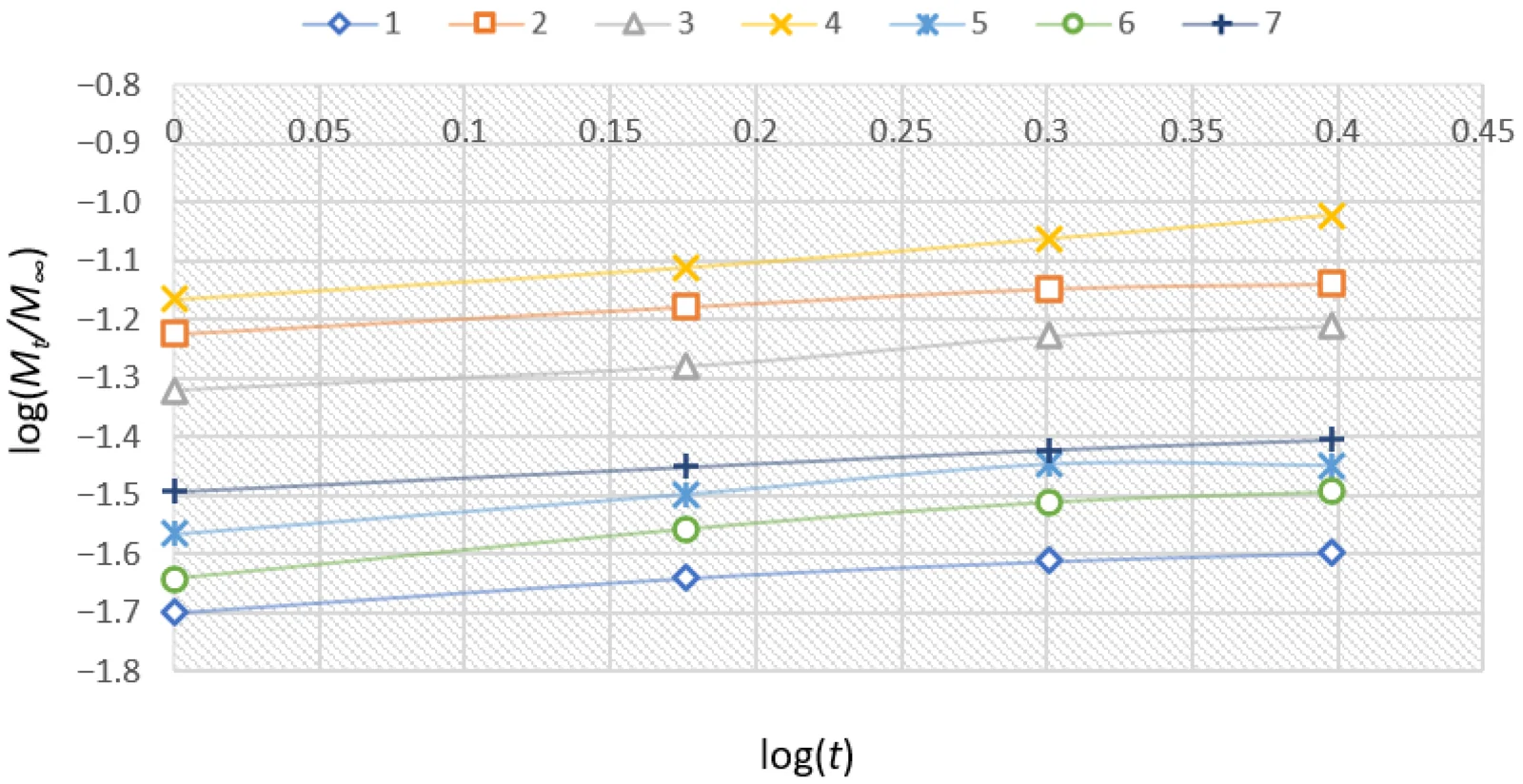
Release kinetic profile according to the Korsmeyer–Peppas model.

The curcumin release kinetics according to the Higuchi and Korsmeyer–Peppas models are presented in Figure 11 and Figure 12, respectively.

The optimal kinetic model was selected based on the Akaike criterion, as shown in Table 3. According to the results presented in Table 3, the Korsmeyer–Peppas model provided the lowest *AICc* value for samples 1–3 and 5–7, whereas the Higuchi model provided the lowest *AICc* value for sample 4. However, *ΔAICc* was below 2 for samples 1, 2, 3, 5, and 6, indicating that, for these samples, the two models cannot be statistically distinguished. Only for samples 4 (*ΔAICc* = 2.55) and 7 (*ΔAICc* = 3.88) was one of the models better supported by the data.

The values of the release exponent (*n*) obtained using the Korsmeyer–Peppas model ranged from 0.2213 to 0.3824, values consistent with a release process controlled primarily by diffusion. Given the small number of experimental data points (*n* = 4), this interpretation should be considered indicative. However, because the investigated systems consisted of polymer coatings deposited on a heterogeneous textile structure rather than ideal matrices with a well-defined geometry, these values should be interpreted with caution. The release behavior may have also been influenced by the ionic crosslinking of alginate with Ca^2+^ ions, which can restrict curcumin mobility within the polymer matrix. Thus, the release profile may involve an initially faster release of the curcumin fraction located at or near the coating surface, followed by slower diffusion of the compound retained within the crosslinked alginate matrix [48,49].

Sample 4, which had the lowest curcumin concentration, was better described by the Higuchi model (*ΔAICc* = 2.55), a finding consistent with curcumin release being primarily controlled by diffusion through the polymer layer. Conversely, the higher curcumin concentrations in samples 2 and 3 may have contributed to a more complex release behavior, better described by the Korsmeyer–Peppas model. This behavior may be associated with a more heterogeneous distribution of curcumin within the polymer matrix, possible curcumin aggregation, and the localization of a fraction of the compound at the coating surface. However, additional structural investigations would be required to confirm the contribution of these factors to the observed release kinetics.

## 4. Conclusions

Bioactive alginate-based dispersions containing curcumin and *Aloe vera* were successfully applied to CaCl_2_-pretreated cotton fabrics, producing coatings with different deposition, color, release, and antibacterial characteristics depending on their composition. Optical microscopy showed a relatively uniform distribution of curcumin in the prepared dispersions, while SEM observations confirmed the presence of polymer deposits and film-like regions on the fiber surfaces. The add-on values ranged from 9.44 to 17.73%, demonstrating that, despite the high water content of the dispersions, a sufficient amount of nonvolatile compounds was deposited and retained on the fabric to produce a substantial increase in fabric weight. The variation in these values suggests that formulation composition influenced the final coating deposition.

Color analysis reflected the contribution of curcumin to the yellow coloration of the coated fabrics. The strong positive correlation between *K/S* and b* confirmed the relationship between color strength and the yellow component, and the absence of a significant correlation between *K/S* and add-on showed that the total mass deposited was not the determining factor for color strength. The curcumin release profiles showed an initially faster release followed by a gradual approach to a plateau. The values of the Korsmeyer–Peppas exponent corresponded to a release primarily via diffusion, although the limited number of experimental data points does not allow for a firm conclusion regarding the mechanism. The Korsmeyer–Peppas model yielded the lowest *AICc* values for samples 1–3 and 5–7, whereas the Higuchi model provided the lowest value for sample 4. However, the small differences between the models for most samples indicate that these results should not be interpreted as evidence of completely distinct release mechanisms.

The antibacterial response was also dependent on formulation composition. Sample 7, containing 1.5% sodium alginate, 0.1% curcumin, and 1% *Aloe vera*, produced the largest inhibition zones against both bacterial strains. Samples 3 and 4 did not produce measurable inhibition zones, but the absence of bacterial growth in the contact area beneath the specimens indicated a satisfactory contact-based effect according to SR EN ISO 20645:2004. Furthermore, the exposure of HaCaT keratinocytes to extracts from all coated textiles did not compromise cell viability or membrane integrity, and the cells maintained their specific morphology. In conclusion, the results show that coatings based on alginate, curcumin, and *Aloe vera* can impart antibacterial activity to textile substrates, provide a gradual release of curcumin during the first few hours, and elicit a favorable in vitro cellular response.

## Figures and Tables

**Table 1:** Composition of the bioactive alginate-based dispersions.

Sample	Sodium Alginate % (*w*/*v*)	Glycerin % (*v*/*v*)	Tween 20 % (*v*/*v*)	Curcumin % (*w*/*v*)	*Aloe vera* % (*w*/*v*)
**1**	1	0.5	0.5	0.1	0.5
**2**	1	0.5	0.5	0.1	1
**3**	1	0.5	0.5	0.05	1
**4**	1	0.5	0.5	0.02	1
**5**	1	0.5	0.5	0.1	1.5
**6**	1.25	0.5	0.5	0.1	1
**7**	1.5	0.5	0.5	0.1	1

**Table 2:** CIELAB parameters, color strength, and Pearson correlation matrix for the functionalized cotton fabrics.

		*K/S (430 nm)*	L*	a*	b*	*Add-on (%)*	
	K/S (430 nm)	1	−0.7659 *	0.61546	0.97579 *	0.5468226	
	L*	−0.765925	1	−0.97642 *	−0.83906 *	−0.51662	
	a*	0.6154595	−0.97642	1	0.714257	0.429271	
	b*	0.9757965	−0.83906	0.714257	1	0.484302	
	Add-on (%)	0.5468226	−0.51662	0.429271	0.484302	1	
	1	2	3	4	5	6	7
*K/S* (430 nm)	6.38	4.64	2.34	1.56	2.88	3.08	2.71
L*	87.44	88.08	90.67	91.56	88.58	88.75	88.03
a*	1.86	1.71	−3.68	−5.09	1.54	0.66	2.55
b*	85.83	80.15	68.25	60.7	71.91	72.76	69.82
	1		0		−1		
	Positive correlation		No correlation		Negative correlation		

* statistically significant correlation (*p* < 0.05). L*—lightness from 0 (black) to 100 (white); −a* and +a* indicate green and red shades, respectively; −b* and +b* indicate blue and yellow shades, respectively; *K/S*—color strength calculated at 430 nm.

**Table 3:** Model-fitting results and *AICc*-based comparison of kinetic models for curcumin release.

Model Parameters	Higuchi			Korsmeyer–Peppas			
						*n*	
*R* ^2^	1	0.9621		1	0.9751	0.2593	
	2	0.9594		2	0.9751	0.2213	
	3	0.9803		3	0.9819	0.2920	
	4	0.9974		4	0.9950	0.3601	
	5	0.9086		5	0.9348	0.3131	
	6	0.9640		6	0.9734	0.3824	
	7	0.9918		7	0.9972	0.2242	
*RSS*	1	0.0062		1	0.0044		
	2	0.0416		2	0.0289		
	3	0.0238		3	0.0232		
	4	0.0105		4	0.0199		
	5	0.0444		5	0.0389		
	6	0.0186		6	0.0160		
	7	0.0025		7	0.0009		
*AIC*	1	−21.8922		1	−23.2880		
	2	−14.2661		2	−15.7265		
	3	−16.4983		3	−16.5966		
	4	−19.7656		4	−17.2173		
	5	−14.0064		5	−14.5323		
	6	−17.4862		6	−18.0800		
	7	−25.5797		7	−29.4589		
*AICc*	1	−9.8922		1	−11.2880		
	2	−2.2661		2	−3.7265		
	3	−4.4983		3	−4.5966		
	4	−7.7656		4	−5.2173		
	5	−2.0064		5	−2.5323		
	6	−5.4862		6	−6.0800		
	7	−13.5797		7	−17.4589		
*ΔAICc*	1	1.3958		1	0.0000		OPTIMAL MODEL
	2	1.4604		2	0.0000		OPTIMAL MODEL
	3	0.0983		3	0.0000		OPTIMAL MODEL
	4	0.0000	OPTIMAL MODEL	4	2.5483		
	5	0.5260		5	0.0000		OPTIMAL MODEL
	6	0.5938		6	0.0000		OPTIMAL MODEL
	7	3.8792		7	0.0000		OPTIMAL MODEL

## Data Availability

The original contributions presented in this study are included in the article. Further inquiries can be directed to the corresponding author.
